# Nosocomial infections and antibiotic resistance pattern in open-heart surgery patients at Imam Ali Hospital in Kermanshah, Iran

**DOI:** 10.3205/dgkh000292

**Published:** 2017-05-24

**Authors:** Fatemeh Heydarpour, Youssef Rahmani, Behzad Heydarpour, Atefeh Asadmobini

**Affiliations:** 1 Cardiovascular Research Center, Kermanshah University of Medical Sciences, Kermanshah, Iran

**Keywords:** open heart surgery, nosocomial infection, SSI, antibiotic resistance, Iran

## Abstract

**Background:** Patients undergoing open heart surgery have a relatively high risk of acquiring nosocomial infections. The development of antibiotic-resistant infections is associated with prolonged hospital stays and mortalities.

**Objectives:** The present study was conducted to investigate nosocomial infections and the antibiotic resistance pattern in bacteria causing these infections in open heart surgery patients at Imam Ali Hospital in Kermanshah in the west of Iran over a 4-year period from March 2011 to March 2014.

**Materials and methods:** The present cross-sectional study was conducted on 135 cases of nosocomial infection among open heart surgery patients. The demographic characteristics and the risk factors of each case of infection were recorded. The antibiotic susceptibility test was carried out using the Minimum Inhibitory Concentration (MIC) method based on the Clinical and Laboratory Standards Institute (CLSI) protocol. The data collected were then analyzed in SPSS-16.

**Results:** Out of the 6,000 patients who underwent open heart surgery during this 4-year period at the selected hospital, nosocomial infections developed in 135 patients (2.25%), 59.3% of whom were female and 40.7% male. Surgery site infection (SSI), pneumonia (PNEU), urinary tract infection (UTI) and blood stream infection (BSI) affected 52.6%, 37%, 9.6% and 0.8% of the cases, respectively. *E.coli*, *Klebsiella* spp. and *S. aureus* were the most common bacteria causing the nosocomial infections. *E. coli* was most frequently resistant to imipenem (23.3%) *Klebsiella* spp. to gentamicin (38.5%) *S. aureus* to co-trimoxazole (54.2%).

**Conclusion:** SSI had a high prevalence in this study. Further studies should therefore be conducted to examine the risk factors associated with SSI in open heart surgery. Various studies have shown that antibiotic resistance patterns are different in different regions. Finding a definitive treatment therefore requires an antibiogram.

## Zusammenfassung

**Hintergrund:** Das Risiko nosokomialer Infektionen ist nach offen herzchirurgischen Eingriffen vergleichsweise hoch. Im Fall einer Infektion durch Antibiotika resistente Bakterien verlängert sich die Aufenthaltsdauer und erhöht sich die Mortalität.

**Zielsetzung:** Von März 2011 bis März 2014 wurden die Häufigkeit nosokomialer Infektionen nach offen herzchirurgischen Eingriffen und die Antibiotikaresistenz der ätiologisch zugrunde liegenden Erreger im Imam Ali Hospital in Kermanshah, Westiran, untersucht.

**Material und Methoden:** Die Querschnittsstudie wurde bei 135 Patienten mit einer nosokomialen Infektion nach offenen herzchirurgischen Eingriffen durchgeführt. Von jedem Patienten wurden die demographischen Daten und Risikofaktoren erfasst. Zur Bestimmung der Antibiotikaempfindlichkeit wurde die minimale Hemmkonzentration (MHK) im Verdünnungstest gemäß Protokoll des Clinical and Laboratory Standards Institute (CLSI) ermittelt. Die Auswertung erfolgte in SPSS-16.

**Ergebnisse:** Von 6.000 Patienten (59,3% weiblich, 40,7% männlich) entwickelten 135 (2,25%) nach offenen herzchirurgischen Eingriffen eine nosokomiale Infektion. Der Anteil von Surgery Site Infections (SSI), Pneumonien, Harnweg- und Blutstrominfektionen betrug 52,6%, 37%, 9,6% bzw. 0,8%. *E. coli*, *Klebsiella* spp. and *S. aureus* waren die häufigsten Erreger. *E. coli* war am häufigsten gegen Imipenem (23,3%) resistent, *Klebsiella* spp. am häufigsten gegen Gentamicin (38,5%) und *S. aureus* am häufigsten gegen Cotrimoxazol (54,2%).

**Schlussfolgerung:** Auf Grund der hohen Prävalenz von SSI sollten weitere Studien zur Abklärung der infrage kommenden Risikofaktoren durchgeführt werden. Da die Antibiotikaresistenz regional differiert, ist parallel zum kalkulierten Therapiebeginn ein Antibiogramm erforderlich, um die Therapie gezielt fortsetzen zu können.

## Introduction

Nosocomial infections are considered a serious threat to patients’ health, especially in intensive care units [1]. About 8.7% of hospitalized patients worldwide develop nosocomial infections. These infections cause surgery failure, organ rejection, treatment failure, increased costs, death due to prolonged hospital stays after infection, and psychological stress in the patients [2], [3], [4]. Nosocomial infections are estimated to be responsible for about 80,000 deaths in the US every year [5]. 

Nosocomial infections can cause serious post-operative complications in patients and significantly increase the likelihood of death; they can also increase the need for antibiotic treatment as well as the economic burden of diseases [6].

Cardiac patients, especially those undergoing heart surgery, appear to be at a greater risk for nosocomial infections due to their multiple surgical wounds, the use of invasive devices after surgery, the common use of antibiotics before surgery, and the longer hospital stays [7].

Reports indicate a 5% to 21% prevalence of nosocomial infections after heart surgery, which can pose a serious health threat to these patients [8], [9]. Moreover, the current trend of antibiotic administration before surgery may lead to the emergence of antibiotic-resistant pathogens in these patients. A major barrier to the treatment of nosocomial infections is the increase in antibiotic-resistant organisms. Preventing nosocomial infections is therefore gaining urgency [10], [11]. 

Knowledge about the common pathogens in every hospital and hospital ward can contribute to the effective control of infections. Measuring the antibiotic resistance of bacteria is an essential task. To the best of the authors’ knowledge, the present study is the first of its kind on heart patients in western Iran that aims to investigate nosocomial infections and antibiotic resistance patterns among the pathogens causing these infections at Imam Ali Heart Hospital in Kermanshah, western Iran. 

## Materials and methods

The present cross-sectional study was conducted on all patients who developed nosocomial infections at Imam Ali Hospital in Kermanshah over a four-year period from March 2011 to March 2014 using the census method. Nosocomial infections were defined according to the national guidelines for nosocomial infection care [12]. Data pertaining to the patients affected by nosocomial infections, including demographic and clinical details, medical history, risk factors, type of infection and their clinical samples were collected and analyzed with SPSS-16. The risk factors defined were diabetes, hypertension, lung diseases, urinary catheter, suction, vein catheter, artery catheter, tracheostomy, and intubation. 

The samples taken from the patients included discharge from infected deep tissue at the site of the surgical wounds, sputum, tracheal discharges, infected tissue debris, infected mediastinal tissue drainage, urine, and blood. The samples were taken by a laboratory technician and an infection control nurse and were immediately transferred to the laboratory. To avoid killing the microorganisms living in the samples, the samples were transferred in a nutritient soybean casein digest medium (Tryptone Soya Broth). The microorganisms causing the infections were detected based on standard guidelines for microbiological examination [13], [14].

The antimicrobial susceptibility test was performed using the standard broth dilution (Micro Dilution Broth) technique. The minimum inhibitory concentration (MIC) was defined as the minimum dose of an antibiotic that prevented the visible growth of bacteria following overnight culture, and was determined based on the CLSI protocol of 2010. A standard suspension, equivalent to McFarland standard of 0.5, was prepared using Mueller Hinton Broth (MHB; Merck, Germany) by employing a swab to remove a number of bacterial colonies from those grown on blood agar and eosin methylene blue (EMB) agar media and dissolving them in sterilized saline. The standard was then placed in a bain-marie for 1–2 hours and cultured on Mueller Hinton agar. The antibiotic discs purchased from the Padtan Teb Company (Tehran, Iran) were then placed on the standard.

## Results

Of the 38,057 patients hospitalized during the four years from March 2011 to March 2014, 427 (1.1%) developed nosocomial infections, 257 (60.2%) of whom were female and 170 (39.8%) were male. A total of 6,000 of the hospitalized patients had open heart surgeries and 135 (2.25%) of them subsequently developed nosocomial infections; 80 of the patients who developed nosocomial infections (59.3%) were female and 55 (40.7%) were male. The mean overall age of the patients who developed nosocomial infections was 65.2 ± 11.83 years, and the mean age of those with heart surgeries who developed nosocomial infections was 69.6 ± 11.97 years (61.7 ± 11.93 years for female and 64.9 ± 99 years for male patients). Surgery Site Infection (SSI) occurred in 71 patients (52.6%), pneumonia in 50 patients (37%), urinary tract infection (UTI) in 13 (9.6%) patients, and blood stream infection (BSI) in one (0.8%) patient. Table 1 (Tab. 1) presents the demographic characteristics and risk factors for infection in the heart surgery patients. Table 2 (Tab. 2) presents the factors causing infection by the site and type of the nosocomial infection; Table 3 (Tab. 3) shows these factors by hospital ward. 

Table 4 (Tab. 4) and Table 5 (Tab. 5) show the antibiotic resistance pattern in the Gram-positive and the Gram-negative bacteria, respectively. The most common bacteria observed over four-year period included *E. coli*, which affected 30 patients (24%), *Klebsiella* spp., affecting 26 patients (21%), and *S. aureus*, affecting 24 patients (19%). The antibiotics to which *E.coli* was most resistant included imipenem in 7 cases (23.3%) and gentamicin and nalidixic acid in 6 cases (20%). The antibiotics to which *Klebsiella* spp. was most resistant included gentamicin in 10 cases (38.5%) and imipenem in 7 cases (27%). *S. aureus* was most resistant to co-trimoxazole with 13 cases (54.2%) and penicillin with 9 (37.5%). 

## Discussion

Despite the great advances in surgical techniques, such as prophylactic antibiotics, disinfection and sterilization, SSIs still cause problems for a large number of patients [15]. The rate of nosocomial infections was 2.25% in the present study, of which 1.18% were SSIs. Various studies have reported nosocomial infections after cardiac surgery to exceed 20% [8], [16], [17], [18], [19], [20]. The incidence of nosocomial infections was reported as 16% in the study by Lomtadze et al. and 8.3% in the study by Davoodi et al. [21], [22]. Microbiological evidence suggests that 5% of open heart surgery patients develop nosocomial infections [9]. Under-reporting appears to be in excess of 50% at Imam Ali Hospital of Kermanshah, and the health authorities need to take measures at the ministerial and regional levels in order to reduce this under-reporting. 

SSI was reported as 1.18% in the present study, but as 3% in the study by Lepelletier et al. and 13.5% by Lee et al. [23], [24]. Other studies have reported the rate of SSI to range from 3% to 10.4% [15], [25], [26], [27]. Davoodi et al. reported this rate as 27.8% [22]. The high percentage of SSI among cardiac surgery patients in the present study may be due to neglecting aseptic surgical techniques and postoperative hygiene.

In this study, diabetes was the main risk factor for SSI, as 42.3% of the infected patients were diabetics. Lee et al. reported this rate as 37.1% [24]. According to studies conducted by Yamashita and Vardakas, diabetic patients are predisposed to a variety of infections, including nosocomial infections [28], [29]. According to Lee et al., however, diabetes is not a significant risk factor for SSI [24]. SSI in the present study was chiefly caused by *E. coli* (26.8%), followed by *S. aureus* and *Klebsiella* spp. (14%). In studies by Davoodi et al., *S. aureus* was the main cause of SSI [22], [30], [31].

The present study reported the rate of pneumonia as 37%, Davoodi et al. reported it as 25.3% and Lomtadze et al. reported it as 7% [21], [22]. In line with the present findings, some of these studies reported intubation respiratory infections as a major risk factor for pneumonia [21], [31]. 

The main causes of respiratory infections were *Klebsiella* spp. and *S. aureus* in the present study; in the study by Davoodi et al., however, *P. aeruginosa* and *S. aureus* were the main bacteria isolated from the cases of pneumonia [22]. The main bacteria causing nosocomial pneumonia included *P. aeruginosa*, *A. baumannii* and Enterobacteriaceae [32].

The present study reported the rate of UTI as 9.6% and found its main risk factor to be urinary catheterization, which is in line with the results of other studies [22], [33], [34]. The most common microorganism causing UTI in the present study was *E. coli*, Enterobacteriaceae in the study by Mirinazhad et al. and *E. coli* in many other studies conducted in Iran [34], [35], [36], [37], [38], [39]. 

The present study reported the rate of BSI 0.8%, Lomtadze et al. reported it as 7.8% and Davoodi et al. as 8.6% [21], [22]. Barker reported the rate of BSI as 2.6% in children after cardiac surgery [40]. The most common microorganisms causing BSI included *E. coli* in the present study, and *E. coli*, *Enterococcus* spp. and *P. aeruginosa* in the study by Al-Hazmi et al. [41].

*E. coli*, *Klebsiella* spp. and *P. aeruginosa* were the most common Gram-negative bacteria and *S. aureus* the most common Gram-positive bacterium isolated in the present study. In Davoodi’s study, *E. coli* and *P. aeruginosa* were the most common Gram-negative bacteria and *S. aureus* the most common Gram-positive bacterium isolated [22]. In a study by Lavakhamseh et al., the most common bacterium isolated from patients with nosocomial infection was *E. coli* [42].

In the present study, *E. coli* showed the highest resistance to imipenem (23.3%), followed by gentamicin (20%) and nalidixic acid (20%). In the study by Davoodi et al., *E. coli* showed the highest resistance to ceftazidime (27.5%) and imipenem (13.8%) [22]. In Lavakhamseh’s study, *E. coli* showed the highest resistance to co-trimoxazole (57.47%) [42].

In the present study, *Klebsiella* spp. showed the highest resistance to gentamicin (38.5%) and imipenem (27%); in Davoodi’s study, it showed the highest resistance to ceftazidime (71.42%), followed by ceftriaxone (57.1%) and ciprofloxacin (57.1%) [22]. In the present study, *S. aureus* showed a high resistance to co-trimoxazole (54.2%) and penicillin (37.5%), and 50% of the cases of *S. aureus* were multidrug resistant. In Davoodi’s study, *S. aureus* was resistant to penicillin in 38.7% of the cases and to co-trimoxazole in 38.7% [22].

*S. aureus* and *S. epidermidis* showed high levels of antibiotic resistance in the present study, with *S. aureus* showing a resistance of 12.5% to vancomycin; in Davoodi’s study, this rate was reported as 3.2%.

Infections caused by antibiotic-resistant pathogens are often associated with a higher mortality rate compared to the same infections caused by antibiotic-sensitive pathogens [43], [44]. Careful infection control strategies should therefore be adopted and antibiotic-resistance patterns in nosocomial infections should be periodically reviewed, especially in cardiac surgery centers, in order to achieve more effective antibiotic treatment for patients. 

## Conclusion

The present study showed that the rate of nosocomial infection following open heart surgery is low and less than that suggested by microbiological evidence. The rate of SSI, however, was shown to be high (1.18%). SSI increases ICU stays by 2–3.7 times [27], [45], [46] and the mortality rate 3.4- to 36.7-fold [25], [45], [46], [47]. Knowledge on how SSI is developed and its risk factors can be highly beneficial for the effective allocation of resources and the reduction of treatment costs. Further studies are therefore recommended on the risk factors associated with SSI and the reasons for the low rates of NIs in this hospital. Various studies suggest that antibiotic resistance patterns are different in different regions. An antibiogram is therefore essential for finding a definitive treatment.

## Notes

### Competing interests

The authors declare that they have no competing interests.

### Acknowledgements

This study was supported by Kermanshah University of Medical Science. The authors are grateful to Mr. Mohammad Reza Forozesh and Miss Zahra Hosseini for their kind assistance in data gathering.

## Figures and Tables

**Table 1:**
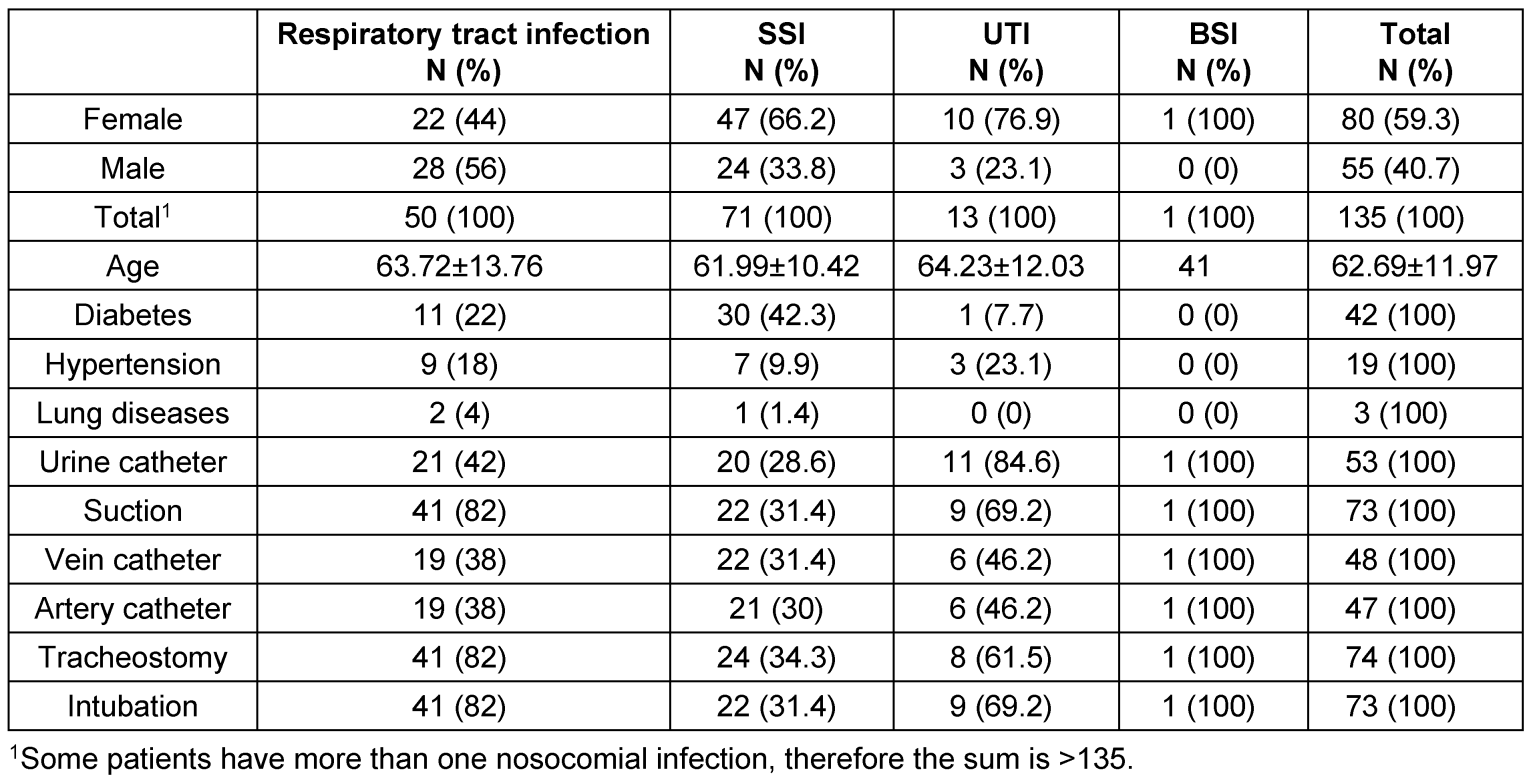
Demographic features and risk factors of infection

**Table 2:**
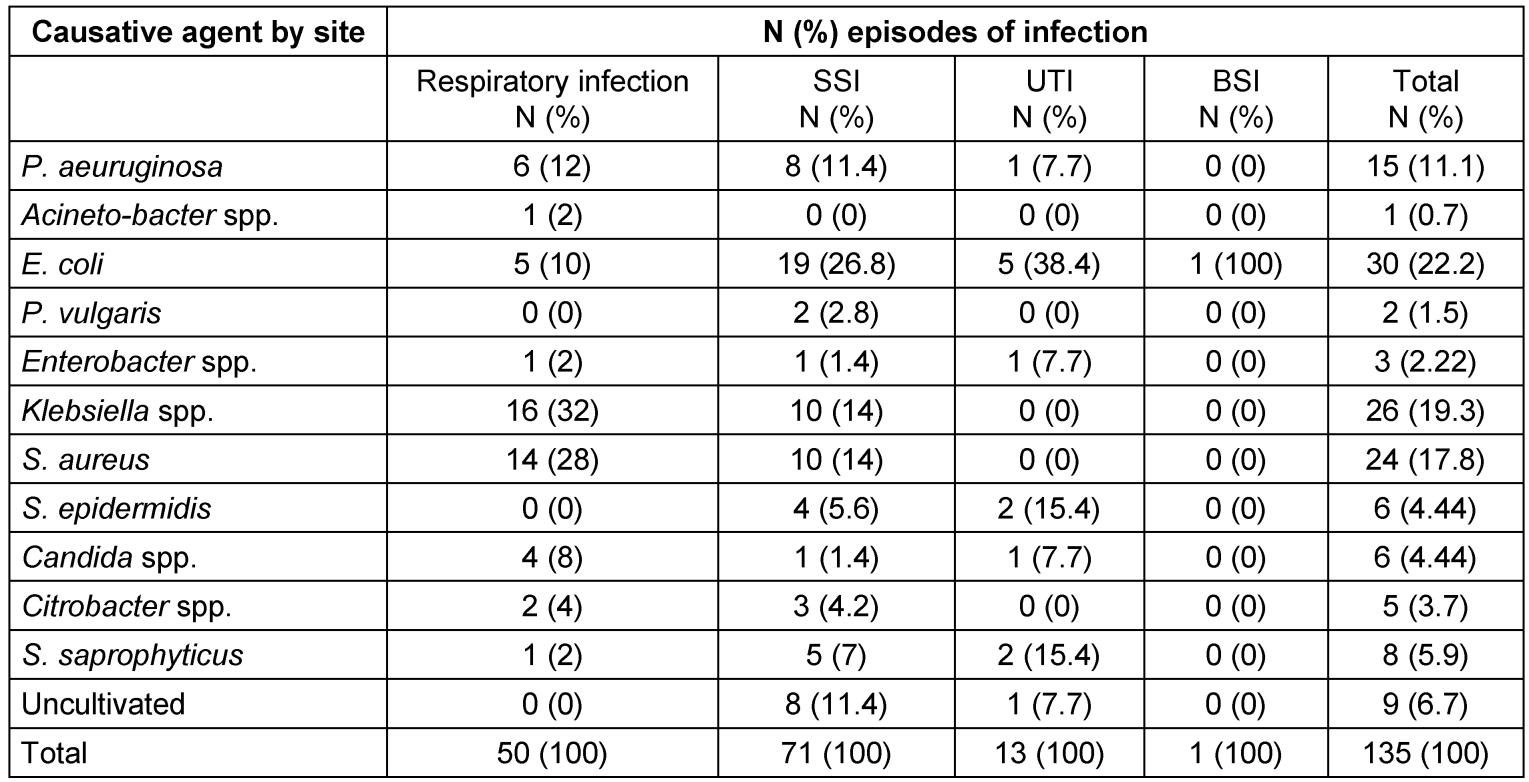
Causative agent of nosocomial infections

**Table 3:**
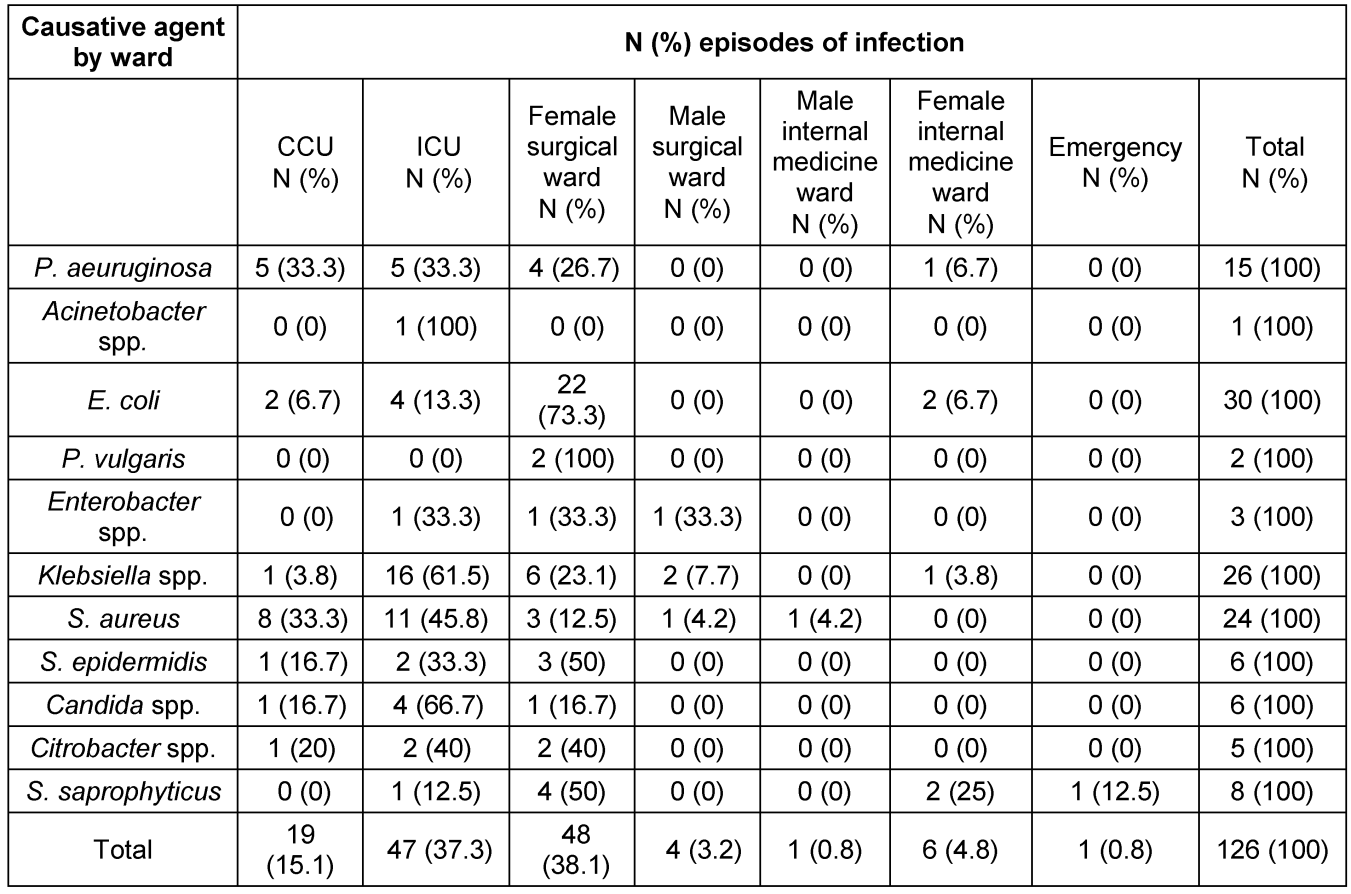
Causative agent of nosocomial infections by ward

**Table 4:**
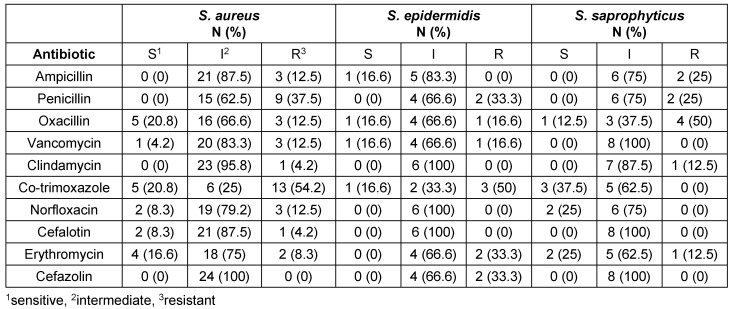
Antibiotic resistance pattern of Gram-positive bacteria isolated from infection

**Table 5:**
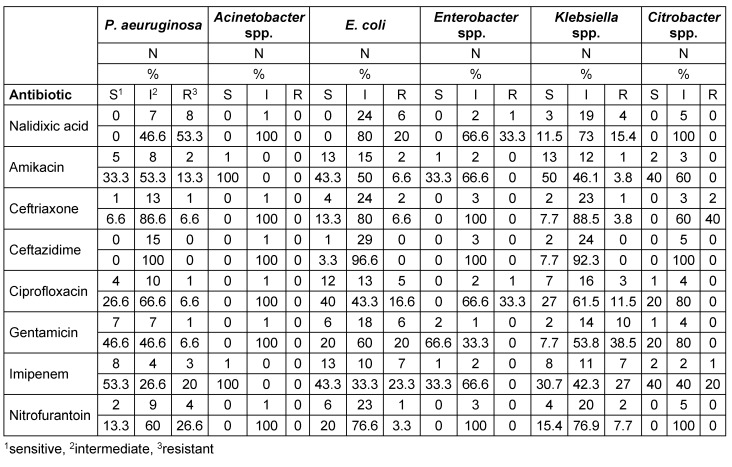
Antibiotic resistance pattern of Gram-negative bacteria isolated from infection
